# Supplementary material for: A Novel Mitovirus PsMV2 Facilitates the Virulence of Wheat Stripe Rust Fungus
Source: Viruses. 2023 May 28;15(6):1265. doi: 10.3390/v15061265 (PMC10301018; doi:10.3390/v15061265)
Supplement: Supplementary file 1 [file viruses-15-01265-s001.zip › viruses-2352489-supplementary.pdf]

Supplementary Table S1. Primers used in this study.

Function	Primer Name	Primer Sequence (5'-3')
RACE	5' RACE-GSP1	CCGACAGAGCCCTTATCTTATT
	5' RACE-GSP2	GAAAGGAGCAGTGGTGTCTTT
	5' RACE-nested GSP	GGATGTTATGATACGAGCCTTT
	3' RACE-GSP1	GCCTATCATCAGTTGGTCTTACTTT
	3' RACE-nested GSP	GGACGTATCATATAGTTCTCGGTATT
Gene expression	PsMV2-T-F	CCCCTGTCCACGTAATGTGGACAACTCCTA
	PsMV2-T-R	GCCCTTGTCCCCAGTAATGGGGACAACGCT
	PsMV2-RdRp-F	CACCTCTCCACTAAGGCG
	PsMV2-RdRp-R	GATTGGGAAAGGGCTATGCT
BSMV-HIGS	BSMV-PsMV2-1as-F	TTTTTAGCTAGCTGATTAATTAAAGGAC CCGATTAAAGCACGA
	BSMV-PsMV2-1as-R	TCCGTTGCTAGCTGAGCGGCCGCAGAAT TCGGAGGGCATCACC
	BSMV-PsMV2-2as-F	TTTTTAGCTAGCTGATTAATTAACCCGTT ATGGGAAGATTCGGT
	BSMV-PsMV2-2as-F	TCCGTTGCTAGCTGAGCGGCCGCATACT CTCTCGCCAGGTCGT
Overexpression in *N. benthamiana*	PsMV2-PVX-RdRp-F	AGGTCAGCACCAGCTAGCATCGATCACCTCTCCACTAAG GCGGG
	PsMV2-PVX-RdRp-R	CTTAACCGTTCATCGGCGGTCGACGATTGGGAAAGGGCT ATGCTCC
	PVX-F	CAATCACAGTGTTGGCTTGC
	PVX-R	GACCCTATGGGCTGTGTTG
qRT-PCR	Q-PsMV2-F	ATCTAACGGCTGCGACTGAC
	Q-PsMV2-R	GCCCTACGGCATACCGAATC
	TaEF1-F	TGGTGTCATCAAGCCTGGTATGGT
	TaEF1-R	ACTCATGGTGCATCTCAACGGACT
	PstEF1-F	TTCGCCGTCCGTGATATGAGACAA
	PstEF1-R	ATGCGTATCATGGTGGTGGAGTGA

Supplementary Table S2. Virus names and viral protein accession numbers used for phylogenetic analysis.

Family and Genus	Virus	RdRp
		Accession No.
Mitoviridae		
Unuamitovirus	Puccinia striiformis mitovirus 2	OQ263177.1
	Cronartium ribicola mitovirus 1	AMQ67414.1
	Sclerotinia sclerotiorum mitovirus 9	AHF48625.1
	Hymenoscyphus fraxineus mitovirus 1	AZG04326.1
	Sclerotinia sclerotiorum mitovirus 15	AHF48631.1
	Puccinia striiformis mitovirus 1	AZY91986.1
	Fusarium circinatum mitovirus 1	AKK23542.1
	Fusarium globosum mitovirus 1	BAQ36629.1
	Fusarium coeruleum mitovirus 1	BAQ36630.1
	Fusarium poae mitovirus 2	UWK02074.1
	Thielaviopsis basicola mitovirus	AAZ95420.1
	Sclerotinia sclerotiorum mitovirus 4	AGC24233.1
	Leptosphaeria biglobosa mitovirus 1	AVZ65960.1
	Ophiostoma mitovirus 4	NP_660179.1

	Helicobasidium mompa mitovirus 1-18	BAD72871.1
Duamitovirus	Sclerotinia sclerotiorum mitovirus 3	AGC24232.1
	Ophiostoma mitovirus 3a	CAA06228.1
	Sclerotinia homoeocarpa mitovirus	AAO21337.1
	Botrytis cinerea mitovirus 1	QLF49185.1
	Ophiostoma mitovirus 3b	CAJ32468.1
	Ophiostoma mitovirus 1a	CAJ32466.1
	Tuber aestivum mitovirus	AEG79311.1
	Ophiostoma mitovirus 1b	CAJ32467.1
	Ophiostoma mitovirus 1c	AGT55876.1
Kvaramitovirus	Ophiostoma mitovirus 7	AGT55877.1
Triamitovirus	Rhizoctonia solani mitovirus 39	QIS79099.1
	Rhizoctonia solani mitovirus 30	QDW65420.1
	Rhizoctonia solani mitovirus 23	QDW65458.1
	Rhizoctonia mitovirus 1 RS002	AHL25281.1
	Geopora sumneriana mitovirus 1	QDM55307.1
	Tuber excavatum mitovirus	AEP83726.1
	Rhizophagus sp. RF1 mitovirus	BAJ23143.2
Narnaviridae	Saccharomyces 20S RNA narnavirus	AAC98925.1
	Saccharomyces 23S RNA narnavirus	UQB84404.1

Supplementary Table S3. Information of BLASTp search results of the RNA-dependent RNA polymerase (RdRp) of Puccinia striiformis mitovirus 1.

Taxon	Viruse Name	Accession	Query Cover (%)	Identity (%)	E-Value
Unuamititovirus	Cronartium ribicola mitovirus 1	AMQ67414.1	99	391/807 (48%)	0
	Puccinia striiformis mitovirus 1	AZY91986.1	81	192/670 (29%)	2e-56
	Helicobasidium mompa mitovirus 1-18	BAD72871.1	79	202/644 (31%)	1e-68
	Sclerotinia sclerotiorum mitovirus 9	AHF48625.1	54	146/442 (33%)	2e-54
	Sclerotinia sclerotiorum mitovirus 15	AHF48631.1	54	157/444 (35%)	1e-59
Duamitovirus	Botrytis cinerea mitovirus 1	QLF49185.1	52	149/468 (32%)	4e-48
	Tuber aestivum mitovirus	AEG79311.1	24	77/220 (35%)	4e-33
	Sclerotinia sclerotiorum mitovirus 3	AGC24232.1	52	138/434 (32%)	1e-36
	Ophiostoma mitovirus 3a	CAA06228.1	51	129/445 (29%)	6e-34
Kvaramitovirus	Ophiostoma mitovirus 7	AGT55877.1	43	87/371 (23%)	3e-23
Triamitovirus	Tuber excavatum mitovirus	AEP83726.1	37	103/305 (34%)	3e-40
	Rhizoctonia mitovirus 1 RS002	AHL25281.1	51	127/425 (30%)	2e-45
	Rhizoctonia solani mitovirus 39	QIS79099.1	37	101/307 (33%)	1e-35
	Rhizoctonia solani mitovirus 23	QDW65458.1	38	102/315 (32%)	1e-38
	Geopora sumneriana mitovirus 1	QDM55307.1	46	116/383 (30%)	1e-37

Supplementary Table S4. RT-PCR detection of PsMV2 in 20 *Pst* samples collected in different regions in China (11 different provinces) and in 10 *Pst* samples from 10 other countries.

Source	Sample Number (Strain)	Detection Rate (%)
Hubei	48-16	100%
Guizhou	47-17	
	GZ-3-1	
	GZ-43-1	

Sichuan	MY-31
	MY-12-2
Henan	LSN-11-6
	LSN-6-10
Yunnan	17-1
	18-7
Shandong	2-3-5
	3-16
Xinjiang	XJ-1
Gansu	GS-1
	GS-2
Qinghai	QH-1
Ningxia	NX-1
	NX-2
Shaanxi	YL-1
	YL-2
Egypt	EGY11
America	US10
Denmark	DEN1
Chile	CHI5
Liechtenstein	LIE13
Australian	AUS14
Turkey	TUR6
Nepal	NEP13
Germany	GER2
Israel	ISR11
